# Uncovering PD-L1 and CD8^+^ TILS Expression and Clinical Implication in Cervical Squamous Cell Carcinoma

**DOI:** 10.1155/2020/8164365

**Published:** 2020-08-20

**Authors:** Jingjing Chen, Ping Gu, Haibo Wu

**Affiliations:** ^1^Department of Pathology, The First Affiliated Hospital of USTC, Division of Life Sciences and Medicine, University of Science and Technology of China, Hefei, Anhui 230036, China; ^2^Intelligent Pathology Institute, Division of Life Sciences and Medicine, University of Science and Technology of China, Hefei, Anhui 230036, China

## Abstract

**Objective:**

To investigate the association between programmed death-ligand 1 (PD-L1) coupled with CD8^+^ tumor-infiltrating lymphocytes (TILS) and the clinicopathological features, along with prognosis of cervical squamous cell carcinoma (CSCC).

**Methods:**

95 patients of CSCC received tumor resection at the Department of Pathology of the First Affiliated Hospital of University of Science and Technology of China (USTC) from 2015 to 2020. Full-automatic immunohistochemistry was applied to measure PD-L1 expression and CD8^+^ TILS density. Our literature deeply assessed the links between PD-L1 expression, clinicopathological features, and the influences of combination of PD-L1 and CD8^+^ TILS (PD-L1^+^/CD8^+^ TILS) on the prognosis of CSCC.

**Results:**

64.21% of CSCC patients (61/95) expressed PD-L1, and PD-L1 expression was related to the Federation of Gynecology and Obstetrics (FIGO) stage, tumor size, invasion depth, differentiation degree, metastasis of lymph node, and vascular invasion (*P* < 0.05). Dramatic correlation between PD-L1 expression and CD8^+^ TILS density was illustrated in CSCC patients (*r* = −0.461, *P* < 0.001). Obvious differences in differentiation degree, FIGO stage, infiltration depth, and lymph node metastasis were shown between patients with PD-L1 coupled with high-density of CD8^+^ TILS and those with PD-L1 coupled with low-density of CD8^+^ TILS (*P* < 0.05). Patients with PD-L1 negative expression exhibited better prognosis compared with those with PD-L1 positive expression (*P* < 0.05). Patients with PD-L1 coupled with high-density of CD8^+^ TILS showed better prognostic status, while those with PD-L1 coupled with low-density of CD8^+^ TILS had worse prognostic condition (*P* < 0.05). Differentiation, metastasis of lymph node, and FIGO stage were substantive impact elements of a CSCC patient's overall survival (OS) by Cox multivariate analysis.

**Conclusions:**

CD8^+^ TILS density is related to PD-L1 expression in carcinoma. PD-L1/CD8^+^ TILS density can be regarded as evaluation for the prognosis of patients with CSCC, providing a new therapeutic target in clinical application.

## 1. Introduction

As one of the malignant carcinomas, cervical squamous cell carcinoma (CSCC) widely occurs in gynecology with a long incubation period. 569,847 of new cases worldwide and 311,365 of mortality was shown in the International Agency for Research on Cancer's latest statistics [1]. Nowadays, cervical cancer is mainly treated by surgery and radiotherapy, supplemented by chemotherapy. Recently, the development of new therapeutics targeting immune checkpoints has renewed interest in the use of immunotherapy in cervical cancer patients. Accompanied by new diagnostic techniques and treatment development, cervical cancer incidence in developed countries has decreased dramatically, but the mortality in developing countries still remains at a high level [2].

Multiple factors and genes were reported to be linked to cervical cancer occurrence and progression. During this process, the individual immune function was greatly affected. Studies have found that programmed cell death-1 (PD-1) on tumor-infiltrating lymphocytes (TILS) can be combined with the programmed death ligand-1 (PD-L1) on tumor cells and then inhibits T cell proliferation and activation, thus inducing immune escape of tumor cells [3–6]. Retard of PD-1/PD-L1-caused T cell activation widely occurred in various carcinomas. In cervical carcinoma, PD-1/PD-L1 expression was also studied in TILS [7, 8]. Compared with endometrial and ovarian adenocarcinoma, PD-L1 positive ratio in the TILS component of CSCC was higher. TILS exhibited importantly in forecasting the results of anti-PD-L1 therapies [9], where the assessment of TILS amount and its functional status could be compensated to the PD-L1 expression.

Karim et al. found that more than 50% of TILS in cervical cancer expressed PD-1 and approximately 19% of tumor cells positively expressed PD-L1. Besides, PD-L1 expression had no direct effect on patients' survival ratio. Nevertheless, patients with a relatively excessive amount of infiltrating regulatory T cells showed better survival rate in PD-L1 positive tumors [10]. As other studies demonstrated, expression levels and patterns were related to survival outcomes. In CSCC patients, the disease-free and disease-specific survival rates with diffuse PD-L1 expression were largely lower than those with marginal PD-L1 expression on the tumor and stroma interface [11]. Similarly, one study revealed that cervical adenocarcinoma patients with tumor-related macrophages of PD-L1 positive expression represented lower disease-specific survival ratio, relative to those with adenocarcinoma with PD-L1 negative tumor-associated macrophages. However, in the opposite studies, PD-L1 expression in pretreated samples or CD8^+^ TILS cell density in advanced cervical cancer was not related to progression-free or overall survival [12].

In this literature, we systematically assessed PD-L1 expression and CD8^+^ TILS in CSCC samples and further analyzed their association with clinical features. Taken together, our findings provided a basis for the prognostic assessment of cervical cancer patients and a newly produced target in immunotherapy of cancer.

## 2. Materials and Methods

### 2.1. Materials

95 cases of CSCC and 30 cases of uterine leiomyomas received surgical resection at the First Affiliated Hospital of the University of Science and Technology of China from May 2015 to April 2016. All patients without accepting any radiotherapy, chemotherapy, and biological treatment prior to surgery were diagnosed by pathological detection and confirmed by reexamination. The age of CSCC patients, with an average of 47.18 (±4.93), was from 27 to 66. As classified by the International Federation of Gynecology and Obstetrics (FIGO) stage, 77 cases were from stage I to II and 18 cases were from stage III to IV. 66 cases retained a high level of differentiation, and 19 cases retained a low level of differentiation. The age of uterine leiomyoma patients, with an average age of 47.13 (±4.97), was from 31 to 64. The survival status of each patient was traced by regular follow-ups in the manner of outpatient visits or telephone until February 1, 2020.

### 2.2. Methods

The surgical specimens were fixed by 10% neutral formalin, dehydrated and dipped in wax, embedded, and sectioned. The fixed samples attached with normal tonsils were indicated as positive control and were separately subjected to hematoxylin-eosin (HE) and IHC staining accordingly. The paraffin-embedded blocks were continuously sliced at 3 *μ*m and subjected to IHC staining of PD-L1 and CD8. The primary monoclonal antibody mouse anti-human PD-L1 (clone 22C3, Batch No. 11098487, Cat. M3653) and the secondary antibody PD-L1 IHC 22C3 phamDx (Batch No. 10153256B, Cat. SK006) were purchased from DAKO. Human CD8 monoclonal antibody (SP57) and enhanced DAB staining reagent and amplification kits were ordered from Roche.

### 2.3. Analysis of Results

#### 2.3.1. Positive PD-L1 Analysis

PD-L1 is positively located in tumor cell membrane and/or cytoplasm, presenting in the form of pale yellow to brown particles. Tumor Proportion Score (TPS) merely detected the PD-L1 expression in tumor cells. TPS indicated the ratio deriving from the number of PD-L1-staining tumor cells divided by the overall tumor cell number multiplied by 100. TPS < 1 is negative, and TPS ≥ 1 is positive [13].

#### 2.3.2. Calculation of CD8^+^ TILS Density

CD8^+^ TILS are situated in the cell membrane and cytoplasm after positive staining, and they were shown as pale yellow to brown coarse particles. CD8^+^ TILS situating inside of tumor nests indicated intraepithelial neoplasm infiltration, while CD8^+^ TILS staying in the adjacent stroma represented peripheral neoplasm infiltration. Three rich areas of CD8^+^ TILS in tumor cells were chosen under a low-magnification microscope to avoid bleeding and/or necrotic lesions. The proportion of positive cells under high-magnification microscope (400x) was calculated and then the average was obtained. The proportion of positive cells < 10% represented a low expression of CD8^+^ TILS, 10%-39% represented moderate expression, and 40%-90% indicated high expression. Patients were classified into low-density and high-density groups in line with the median value of CD8^+^ TILS [14]. CD8^+^ TILS groups were further subdivided into four groups in view of the PD-L1 expression, including the PD-L1^+^/CD8^+^ TILS high-density group (group A), PD-L1^+^/CD8^+^ TILS low-density group (group B), PD-L1^−^/CD8^+^ TILS high-density group (group C), and PD-L1^−^/CD8^+^ TILS low-density group (group D). The prognostic details of CSCC patients in the four groups were assessed.

### 2.4. Statistical Analysis

SPSS 26.0 statistical software was applied for data processing. All counted data was represented as the number and rate (%). The comparison existing in compared groups was analyzed by the *χ*^2^ test, and the correlation among variables was added up using Spearman's correlation. Multifactor analysis was performed by proportional hazard regression model. *P* value < 0.05 indicated a significant difference.

## 3. Results

### 3.1. To Analyze PD-L1 and CD8 Expression in Cervical Cancer Tissue

**Figure 1:**
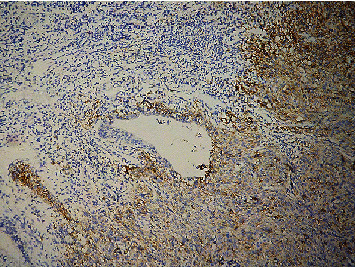
Microscopical image of PD-L1 protein expression in poorly differentiated CSCC (×100) (EnVision assay).

**Figure 2:**
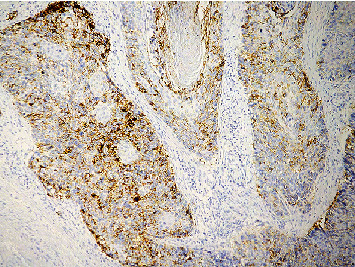
Microscopical image of PD-L1 protein expression in highly moderately differentiated CSCC (×100) (EnVision assay).

**Figure 3:**
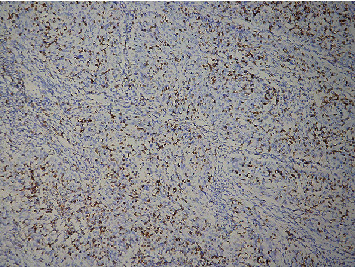
Microscopical image of PD-L1 protein expression in a high-density group of CD8^+^ TILS in CSCC (×100).

**Figure 4:**
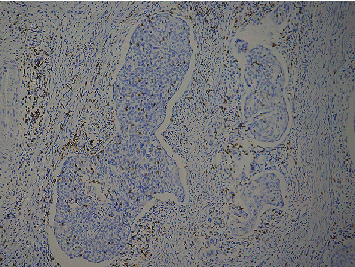
Microscopical image of PD-L1 protein expression in a low-density group of CD8^+^ TILS in CSCC (×100).

Cervical cancer cells and TILS primarily expressed PD-L1 rather than normal cervical tissue. The positive cells expressing PD-L1 were distributed in the form of scatter or aggregated small pieces (Figures 1 and 2). CD8^+^ T lymphocytes, expressed in normal cervical tissue and CSCC, are localized in the TILS membrane (Figures 3 and 4). PD-L1 positive expression ratio in CSCC tissue was 64.21% (61/95), while that of PD-L1 in normal cervical tissue was 6.67% (2/30) (*P* < 0.05). High-density of CD8^+^ TILS in CSCC tissue was 50.53% (48/95), whereas low-density of CD8^+^ TILS attained 49.47% (47/95).

### 3.2. To Analyze PD-L1 Expression and CD8 and the Links between the Density of PD-L1/CD8^+^ TILS and the Clinical Pathological Profile

Cervical cancer patients, with FIGO stages III-IV, tumor diameter ≥ 3 cm, deep muscle invasion, poor differentiation, lymph node metastasis, and vascular invasion (group A for short) had higher PD-L1 positive expression proportion in comparison with those with FIGO stages I-II, moderately high differentiation, tumor diameter < 3 cm, superficial muscular infiltration, nonmetastasis of lymph node, and vascular invasion (group B for short, *χ*^2^ = 8.741, 10.94, 12.41, 4.09, 15.72, and 6.144, *P* < 0.05). PD-L1 positive expression rate in cervical cancer specimens of patients with different ages had no obvious difference (*P* > 0.05). Obvious difference was displayed between group A and group B. CD8 expression in TILS of cervical cancer patients with different ages, lesion size, and vascular invasion presented different degree, and there was no obvious difference (*P* > 0.05). Dramatic difference was demonstrated between the low-density group of PD-L1^+^/CD8^+^ TILS, accounting for 73.7% (14/19) of poorly differentiated tumor cases, and the high-density group of PD-L1^+^/CD8^+^ TILS, taking up 10.5% (2/19) (*P* < 0.05) (Table 1). Evident difference was displayed between the low-density group of PD-L1^+^/CD8, reaching 83.3% (15/18) of stages III to IV cases, and the high-density group of PD-L1^−^/CD8, accounting for 5.6% (1/18) of stages III to IV cases (*P* < 0.05). The ratio of the low-density group of PD-L1^+^/CD8 in poorly differentiated cases was 73.7% (14/20), while that of the high-density group PD-L1^−^/CD8 was 10.5% (2/19). Evident difference was shown in the above two groups (*P* < 0.05). The low-density group of PD-L1^+^/CD8 constituted 73.1% (19/26) over the total cases with lymph node metastasis, whereas the high-density group of PD-L1^−^/CD8 took up 3.8% (1/26) over all cases with lymph node metastasis. The difference was obvious (*P* < 0.05). The ratio of the low-density group of PD-L1^+^/CD8^+^ TILS in deep muscle infiltration cases was 47% (31/66), and that of the high-density group of PD-L1^−^/CD8 was 21.1% (14/66). A significant difference existed in the above two compared groups (*P* < 0.05). Our data indicated that no association was shown between PD-L1/CD8^+^ TILS density and ages and tumor size, along with vascular invasion (*P* > 0.05) (Table 2).

### 3.3. To Assess the Correlation between PD-L1 and CD8^+^ TILS and Analysis of the Impact of Survival Factors

**Figure 5:**
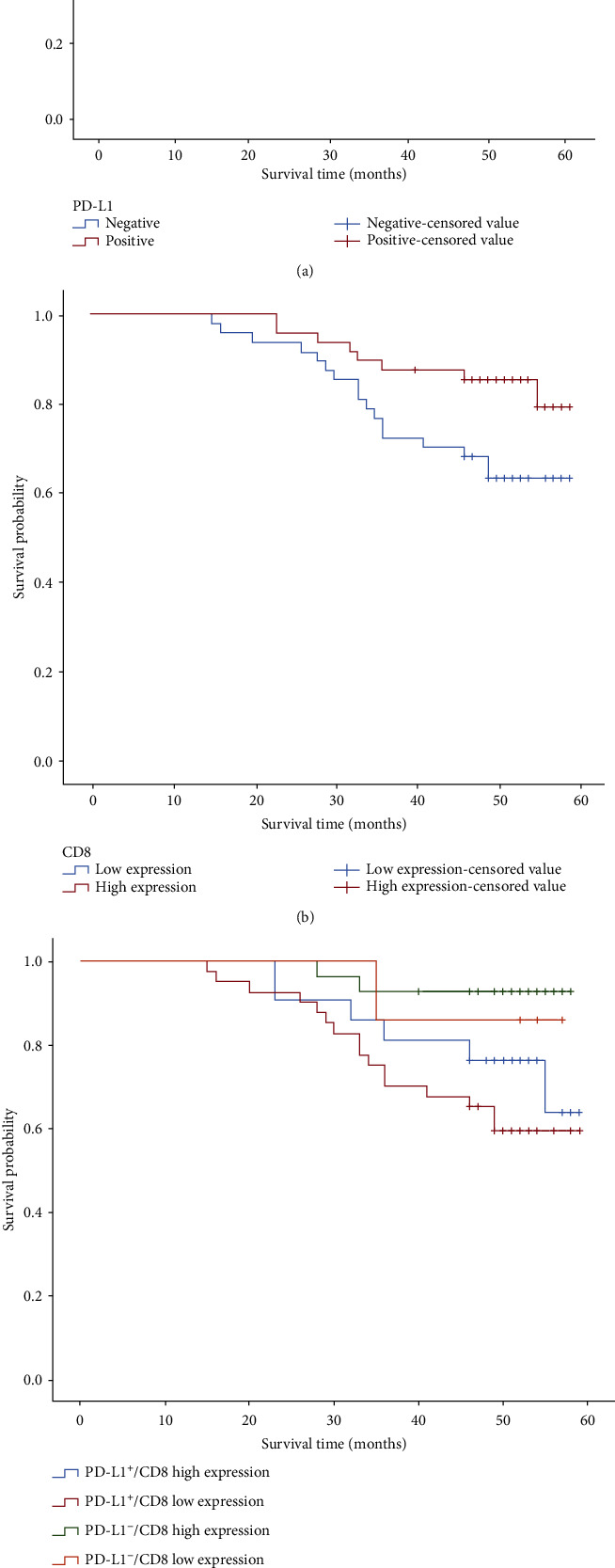
Survival analysis of patients with different immunotypes based on CD8^+^ TILS density and PD-L1 expression in tumors. (a) The influence curve of PD-L1 expression on OS of CSCC patients. (b) The influence curve of CD8^+^ TILS density on OS of CSCC patients. (c) The influence curve of PD-L1/CD8^+^ TILS density on OS of CSCC patients.

In tissues of CSCC, the expression proportion in the CD8^+^ TILS high-density group was 50.53% (48/95), and that in the low-density group of CD8^+^ TILS was 49.47% (47/95). Among the cases with a low expression of CD8^+^ TILS, 65.6% (40/61) of patients positively expressed PD-L1. Negative correlation was revealed between PD-L1 expression and CD8^+^ TILS density (*r* = −0.461, *P* < 0.001). Usually, the median period of follow-up persisted for approximately 29 months (2 to 55 months) before February 2020. 1 case was lost to follow-up, 25 cases died during the period of follow-up, and the 5-year survival rate was 73.68%. Kaplan-Meier analysis showed that PD-L1 negative expression patients exhibited a good prognostic status when a comparison was made with PD-L1 positive expression patients. CD8^+^ TILS high-density patients displayed a good prognosis than the CD8^+^ TILS low-density groups. The high-density group of PD-L1^−^/CD8^+^ TILS had a better prognosis, and the low-density group of PD-L1^+^/CD8^+^ TILS showed a worse prognosis (Figure 5, *P* < 0.05). FIGO stages III to IV, poor differentiation, and metastasis of the lymph node were unattached elements of the CSCC patient's OS utilizing multivariate analysis (Table 3).

## 4. Discussion

PD-L1, also named by B7 homologous protein and CD274, was firstly uncovered in 1999 and located on chromosome 9q24. PD-L1, belonging to the CD28^+^ cytotoxic T-lymphocyte associated protein 4 (CTLA-4) receptor subfamily, is an extremely important costimulatory molecule in immune response, and it displays a leading role in inducing immune tolerance in the microenvironment of the tumor [15]. PD-L1 is widely expressed in immune cells and multiple cancer cells [16]. PD-1, as PD-L1 receptor, is widely expressed in TILS, B cells, natural killer cells, monocytes, and dendritic cells. Currently, PD-1/PD-L1 is a representative immune checkpoint inhibitor, and its function involved in the signaling pathway is reversible. Upon occurrence of PD-L1 binding to PD-1, PD-1/PD-L1 phosphorylated ITSM and ITIM in the intracellular region of T lymphocytes, activating RAS and phosphatidylinositol-3-kinase (PI3K)/protein kinase B protein kinase B (PKB) signaling pathways, inhibiting the activation of downstream T lymphocytes and immune microenvironment of the tumor, and promoting the proliferation and tumor cell escape. Once PD-1/PD-L1 interaction was hindered, T-lymphocyte immune function would be heightened in the tumor, which was the mechanism of the current generation of immunotherapy. Not only could it ameliorate the poorly prognostic status of advanced malignant melanoma, but it also exhibited expected prospects in other solid tumor treatment, being considered as a revolutionary progress in cancer treatment [17]. PD-L1, being a tumor immunotherapy target, performed very well in clinical trials.

Emerging studies demonstrated that PD-L1 expression in CSCC and vulvar squamous cell carcinoma (VSCC) was increased [18]. However, little is known with regard to the association between PD-L1/CD8^+^ TILS density and clinical features. 95 newly emerging cervical cancer patients without adjuvant chemotherapy before surgery were selected and subjected to detect PD-L1 (22C3) and CD8 (SP57). The data showed that PD-L1 was primarily expressed in cervical cancer cells and TILS, and the PD-L1 positive expression rate in cervical cancer tissue was largely higher, compared to that in normal cervical tissue. Moreover, PD-L1 expression in cervical cancer was linked to the size of tumor, invasion of muscle, degree of differentiation, FIGO stage, and vascular invasion. Higher PD-L1 positive expression rate was shown in patients with FIGO stages III to IV, tumor diameter ≥ 3 cm, deep muscle invasion, poor differentiation, lymph node metastasis, and vascular invasion of cervical cancer patients in cervical cancer tissue, compared with that in patients with FIGO stages I to II, moderate and high differentiation, tumor diameter < 3 cm, superficial infiltration of the muscle layer, nonmetastasis of the lymph node, and vascular invasion, implying that PD-L1 positive expression had a connection with the increased tumor infiltration. Ozlen et al. found that PD-L1 expression was linked to the size of the tumor and the lymphatic metastasis. In addition, CD8^+^ TILS expression density in the tumor tissue matched the PD-L1 level [7]. Nevertheless, PD-L1 expression had no association with the overall survival rate of patients. As for the tumor immune checkpoint treatment, PD-L1 was both a target and an immunomodulatory agent, functioning through regulating the immune function of T cells. In the assessment of cervical cancer, PD-L1 expression with CD8^+^ TILS density was combined in the tumor to evaluate the immune environment and the prognostic status of patients. The results showed that negative correlation was between PD-L1 expression and CD8^+^ TILS expression density in tumors (*r* = −0.461, *P* < 0.001), which was similar to Burrack et al.'s report that PD-L1 expression was linked to CD8^+^ TILS density in pancreatic cancer tissues. The results from Burrack et al. probably mirrored the immune response and immunogenicity of tumors [19]. One of the ways of the individual's immune response to tumor antigens is to recruit cytotoxic T cells to secrete interferon-*γ* (IFN-*γ*) in the tumor microenvironment through induction of the JAK/STAT1/interferon regulatory factor-1 (IRF-1) pathway, thus inducing PD-L1 expression [20, 21]. CD8^+^ T cells are cytotoxic T lymphocytes that directly target cancer cells and display an important role in antitumor immunity. Previous studies have shown that the density of CD8^+^ TILS is related to the long-term survival rate of patients with various cancers [22, 23]. Considering that, we performed immune scoring method as Galon et al. described [24] to analyze the PD-L1 expression level in tumor cells and the density of CD8^+^ TILS. Our results indicated that there was a significant correlation between upregulated PD-L1 and high-density CD8^+^ TILS in the tumor center area (*r* = −0.461, *P* < 0.001). Regarding the correlation between PD-L1 and CD8^+^ TILS, the evident association between the CD8^+^ TILS expression density and PD-L1 expression on tumor cells resulted from the mechanism of PD-L1/PD-1, which inhibited T cell activation by repressing transduction of the TCR signal and costimulation of CD28-CD80. Our data proved that PD-L1 overexpression in cervical cancer tissue would generate immune resistance of T cell inactivation in the tumor microenvironment. Our data implied no association between PD-L1/CD8^+^ TILS density groups and ages, tumor size, and vascular invasion (*P* > 0.05), in line with El-Gwendy et al.'s results [25].

The relationship between the PD-L1 expression and the CD8^+^ TILS infiltration density, as well as prognostic status in multiple solid tumors, is controversial. Combination of PD-L1 and CD8^+^ TILS in gastric cancer specimens is of great significance to evaluate the immune status and prognosis of gastric cancer [26], showing to be related to the low survival rate of esophageal cancer, gastric cancer, colorectal cancer, and lung cancer [27, 28]. PD-L1 expression on immune cells was considered as an ideal prognostic factor for vulvar squamous cell carcinoma (SCC) [29] and exerted no effects on the prognosis of laryngopharyngeal squamous cell carcinoma [30]. Kaplan-Meier analysis revealed that patients with PD-L1 negative expression in tumor tissue performed better prognosis than those with PD-L1 positive expression. Patients with high-density CD8^+^ TILS in tumors displayed better prognosis than those with low-density CD8^+^ TILS. The best prognosis of patients was demonstrated in the PD-L1^−^/CD8^+^ TILS high-density group, and the worst prognosis of patients was indicated in the PD-L1^+^/CD8^+^ TILS low-density group. FIGO stages III to IV, poor differentiation, and lymph node metastasis were independent impact factors of CSCC patient's OS after analysis of multivariate Cox regression. Saglam et al. revealed that CD8^+^ T cell density in lowly differentiated CSCC tissues had positive correlation with PD-L1 expression. In our study, multivariate analysis indicated that the high density of CD8^+^ TILS in highly differentiated pericarcinoma was correlated with better survival. PD-L1 expression and CD8^+^ TILS density perhaps contributed to determine the subgroups of patients with a relatively ideal prognosis in poorly differentiated CSCC [31], different from what Saglam et al. revealed. We inferred that the different IHC results of PD-L1 probably resulted from the PD-L1 antibody types used in the respective study and the lack of a universal staining method and evaluation standard. Our procedure referred to the reports of KEYNOTE-028 described in 2017 [13]. Briefly, more than 5% of the PD-L1 expression was defined as the positive threshold. PD-L1 was mostly coexpressed in the membrane and cytoplasm but few in the membrane. The key point of the experiment was aimed at acquiring suitable patients with treatment-related detection indicators, thus obtaining standardized evaluated results and then benefiting from the immunotherapy of patients.

However, several limitations remain in this study. On the one hand, our study merely detected CD8^+^ TILS density in the tumor, but there was no analysis of CD8^+^ TILS density in the interstitial and PD-L1 expression level in lymphocytes, which might be related to the response of immune checkpoint inhibitors. On the other hand, further efforts should be spared to establishing a comprehensive, multifactor immune core evaluation system. Not only can the improved system accurately forecast the prognosis but it can also facilitate clinicians to evaluate the effectiveness of immunotherapy and conduct more effective treatments for cervical cancer.

All in all, CD8^+^ TILS density is related to PD-L1 expression in carcinoma. PD-L1 expression with CD8^+^ TILS density in CSCC has important clinical significance for the diagnosis, treatment, and prognosis of patients. Meanwhile, it can be considered as an evaluation for the prognosis of CSCC and provide personalized treatment for CSCC patients.

## Figures and Tables

**Table 1:** The links between PD-L1 and CD8+ TILS expression in CSCC and clinical pathological profile.

Clinicopathological features	PD-L1 in tumor cell			Intraepithelial CD8^+^ TILS		
	Positive (≥1%), *n* (%)	Negative (<1%), *n* (%)	*P*	Highly expressed (≥17.6/HPF), *n* (%)	Lowly expressed (<17.6/HPF), *n* (%)	*P*
Age (years)						
≤47	32 (62.7)	19 (37.3)	0.750	30 (58.8)	21 (41.2)	0.083
>47	29 (65.9)	15 (34.1)		18 (40.9)	26 (59.1)	
Tumor size (cm)						
<3 cm	25 (49.0)	26 (51.0)	0.001	28 (54.9)	23 (45.1)	0.361
≥3 cm	36 (81.8)	8 (18.2)		20 (45.5)	24 (54.5)	
Interstitial infiltration						
<1/2	11 (37.9)	18 (62.1)	0.001	15 (51.7)	14 (48.3)	0.878
≥1/2	50 (75.8)	16 (24.2)		33 (50.0)	33 (50.0)	
FIGO stage						
I~II	44 (57.1)	33 (42.9)	0.003	45 (58.4)	32 (41.6)	0.002
III~IV	17 (94.4)	1 (5.6)		3 (16.7)	15 (83.3)	
Degree of differentiation						
Moderately high differentiation	45 (59.2)	31 (40.8)	0.043	44 (57.9)	32 (42.1)	0.004
Low differentiation	16 (84.2)	2 (15.8)		4 (21.1)	15 (78.9)	
Vascular invasion						
No	25 (52.1)	23 (47.9)	0.013	23 (47.9)	25 (52.1)	0.609
Yes	36 (76.6)	11 (23.4)		25 (53.2)	22 (46.8)	
Lymph node metastasis						
No	36 (52.2)	33 (47.8)	0.001	41 (59.4)	28 (40.6)	0.005
Yes	25 (96.2)	1 (3.8)		7 (26.9)	19 (73.1)	

**Table 2:** The relationship between PD-L1/CD8^+^ TILS density groups and clinicopathological features.

Clinicopathological features	*n*	A group	B group	C group	D group	*P*
Age (years)						
≤47	51	13 (25.5)	19 (37.3)	17 (33.3)	2 (3.9)	0.473
>47	44	8 (18.2)	21 (47.7)	10 (22.7)	5 (11.4)	
Tumor size (cm)						
≤3	51	7 (13.7)	18 (35.3)	21 (41.2)	5 (9.8)	0.473
>3	44	14 (31.8)	22 (50.0)	6 (13.6)	2 (4.5)	
Depth of interstitial infiltration (cm)						
<1/2	29	2 (6.9)	9 (31.0)	13 (44.8)	5 (17.2)	0.001
≥1/2	66	19 (28.8)	31 (47.0)	14 (21.2)	2 (3.0)	
FIGO stage						
I~II	77	19 (24.7)	25 (32.5)	26 (33.8)	7 (9.1)	0.001
III~IV	18	2 (11.1)	15 (83.3)	1 (5.6)	0 (0.0)	
Degree of differentiation						
Moderately high differentiation	76	19 (25.0)	26 (34.2)	25 (32.9)	6 (7.9)	0.001
Low differentiation	19	2 (10.5)	14 (73.7)	2 (10.5)	1 (5.3)	
Vascular invasion						
No	48	7 (14.6)	18 (37.5)	16 (33.3)	7 (14.6)	0.918
Yes	47	14 (29.8)	22 (46.8)	11 (23.4)	0 (0.0)	
Lymph node metastasis						
No	69	15 (21.7)	21 (30.4)	26 (37.7)	7 (10.1)	0.006
Yes	26	6 (23.1)	19 (73.1)	1 (3.8)	0 (0.0)	

**Table 3:** Multivariate analysis of COX regarding OS in CSCC patients.

Parameters	OS	
	HR (95% CI)	*P*
Size (≤3 cm vs. >3 cm)	0.680 (0.287~1.615)	0.382
Degree of differentiation (moderately high differentiation vs. low differentiation)	0.369 (0.155~0.879)	0.024
Depth of infiltration (superficial muscular layer vs. deep muscular layer)	0.327 (0.073~1.466)	0.144
Lymph node metastasis (metastasis vs. nonmetastasis)	0.220 (0.091~0.534)	0.001
FIGO stage (I~II vs. III~IV)	0.331 (0.145~0.759)	0.009

*P* < 0.05, significant difference; HR: hazard ratio; CI: confidence interval; OS: overall survival.

## Data Availability

The datasets used and/or analyzed during the current study are available from the corresponding author on reasonable request.
